# The interaction between C5a and sphingosine-1-phosphate in neutrophils for antineutrophil cytoplasmic antibody mediated activation

**DOI:** 10.1186/ar4604

**Published:** 2014-07-07

**Authors:** Jian Hao, Yi-Min Huang, Ming-Hui Zhao, Min Chen

**Affiliations:** 1 Renal Division, Department of Medicine, Key Laboratory of Renal Disease, Ministry of Health of China, Key Laboratory of Chronic Kidney Disease Prevention and Treatment (Peking University), Ministry of Education, Peking-Tsinghua Center for Life Sciences, Peking University, First Hospital, Peking University Institute of Nephrology, Beijing 100034, China; 2 Renal Division, Department of Medicine, The Affiliated Hospital of Inner Mongolia Medical University, Huhehot, Inner Mongolia 010050, China

## Abstract

**Introduction:**

C5a plays an crucial role in antineutrophil cytoplasmic antibody (ANCA)-mediated neutrophil recruitment and activation. The current study further investigated the interaction between C5a and sphingosine-1-phosphate (S1P) in neutrophils for ANCA-mediated activation.

**Methods:**

The plasma levels of S1P from 29 patients with ANCA-associated vasculitis (AAV) in active stage and in remission were tested by enzyme-linked immunosorbent assay (ELISA). The generation of S1P was tested in C5a-triggered neutrophils. The effect S1P receptor antagonist was tested on respiratory burst and degranulation of C5a-primed neutrophils activated with ANCA.

**Results:**

The plasma level of circulating S1P was significantly higher in patients with AAV with active disease compared with patients in remission (2034.2 ± 438.5 versus 1489.3 ± 547.4 nmol/L, *P* < 0.001). S1P can prime neutrophils for ANCA-induced respiratory burst and degranulation. Compared with non-triggered neutrophils, the mean fluorescence intensity (MFI) value for CD88 expression was up-regulated significantly in S1P-triggered neutrophils. S1P receptor antagonist decreased oxygen radical production in C5a primed neutrophils induced by ANCA-positive IgG from patients. Blocking S1P inhibited C5a-primed neutrophil migration.

**Conclusions:**

S1P triggered by C5a-primed neutrophils could further activate neutrophils. Blocking S1P could attenuate C5a-induced activation of neutrophils by ANCA. The interaction between S1P and C5a plays an important role in neutrophils for ANCA-mediated activation.

## Introduction

Antineutrophil cytoplasmic autoantibody (ANCA) is closely associated with systemic small vessel vasculitis characterized by segmental vessel wall necrotizing inflammation and a paucity of immunoglobulin deposition [1]. Patients with ANCA-associated vasculitis (AAV) can on the basis of clinical and pathological features be classified as granulomatosis with polyangiitis (GPA, previously named Wegener’s granulomatosis), microscopic polyangiitis (MPA) and eosinophilic granulomatosis with polyangiitis (EGPA, previously named Churg-Strauss syndrome). ANCAs against either proteinase-3 (PR3) or myeloperoxidase (MPO) are hallmarks of AAV [2].

Cumulating evidence suggests that ANCA-induced neutrophil activation plays a crucial role in the pathogenesis of AAV [3-7]. In an anti-MPO antibody-induced mouse vasculitis model, ANCAs are proven to be pathogenic [8]. Furthermore, neutrophils are the primary effector cells in AAV [8,9].

Recent studies, both in the mouse model and in humans, demonstrated that complement activation via the alternative pathway is indispensable in the development of AAV [10-14]. Schreiber *et al*. further found that recombinant C5a dose-dependently primes neutrophils for ANCA-induced respiratory burst. As such, the interaction between C5a and the neutrophil C5a receptor (C5aR, namely, CD88) compose an amplification loop, and is one of the central contributing factors in ANCA-mediated neutrophil recruitment and activation [15].

Regardless of the fact that C5a-C5aR axis may represent an attractive target for immunosuppressive therapy, little is known about the intracellular molecular mechanisms responsible for the C5a-triggered physiological events or key molecules in ANCA-mediated activation of C5a-primed neutrophils [16].

It has become clear that sphingolipids are sources of important signaling molecules [17]. Sphingosine-1-phosphate (S1P) is a potent bioactive sphingolipid metabolite that regulates diverse cellular processes that are important for inflammation and immune responses. Various activated plasma-membrane receptors, such as the platelet-derived growth factor (PDGF) receptor [17,18], FcϵRI and FcγRI antigen receptors [19], tumor necrosis factor receptor 1 (TNFR1) [20-22] and N-formyl-methionyl-leucyl-phenylalanine (FMLP) receptor [23], respectively upregulate sphingosine kinase (Sphk) and generate S1P. S1P is not only an agonist of five specific G protein-coupled S1P receptors (S1PR1-5) that activate diverse downstream signaling pathways, but also has important intracellular (second messenger) actions [22,24-26]. It had previously been demonstrated that S1P plays an important role in autoimmune diseases, such as rheumatoid arthritis, inflammatory bowel disease, multiple sclerosis and asthma [21,22,27-29]. In addition, Sphk1 plays the crucial role in regulating the balance between expression of CD88 and C5a receptor-like 2 (C5L2) in endotoxin-induced lung inflammatory injury [30]. Antisense knockdown of sphk1 in human macrophages inhibits C5a receptor-dependent signal transduction [31].

Therefore, we hypothesized that S1P triggered by C5a-primed neutrophils could further activate neutrophils; the interaction between S1P and C5a might be involved in ANCA-mediated neutrophils respiratory burst and degranulation.

## Methods

### Patients and blood samples

Plasma samples from 29 consecutive patients with active AAV at initial onset, diagnosed at Peking University First Hospital from 2010 to 2011, were collected before immunosuppressive treatment. All these patients met the Chapel Hill Consensus Conference (CHCC) nomenclature of AAV [2]. Patients with secondary vasculitis or with comorbid renal diseases, such as anti-glomerular basement membrane (GBM) nephritis, were excluded. All the above-mentioned 29 patients received corticosteroids and cyclophosphamide for the induction therapy and achieved remission. Plasma samples from these patients at the remission stage were also collected at their regular ambulatory visits. The time of sampling was 11.5 ± 3.0 months after remission was achieved. When sampling at remission, all of them still received oral azathioprine for maintanence therapy. Twenty-nine age- and gender-matched healthy blood donors were enrolled as normal controls. The blood samples from patients and controls were drawn into ethylene diamine tetraacetic acid (EDTA) tubes and put on ice immediately. The blood samples were centrifuged at 2000 g for 15 minutes at 4°C within 30 minutes after collection and the plasma samples were stored at -70°C until use. Disease activity of AAV was assessed according to the Birmingham vasculitis activity score (BVAS) [32]. Remission was defined as ‘absence of disease activity attributable to active disease qualified by the need for ongoing stable maintenance immunosuppressive therapy’, as described previously [33].

### Preparation of immunoglobulin (Ig)G

Normal IgG and ANCA-positive IgG were prepared from plasma of normal volunteers and patients with active MPO-ANCA- or PR3-ANCA-positive primary small vessel vasculitis, using a High-Trap-protein G column on an AKTA-FPLC system (GE Biosciences, San Francisco, CA, USA). None of these patients had dual positivity of PR3-ANCA and MPO-ANCA. Preparation of IgG was performed according to the methods described previously [34,35]. Containers and solution for IgG preparation did not contain lipopolysaccharide (LPS). The concentration of LPS in ANCA-positive IgG was below 0.1 ng/ml.

### Neutrophil isolation

Neutrophils were isolated from heparinized venous blood from healthy donors by density gradient centrifugation on Lymphoprep (Nycomed, Oslo, Norway). Erythrocytes were lysed with ice-cold ammonium chloride buffer, and neutrophils were washed in Hank’s balanced salt solution (HBSS) without Ca^2+^/Mg ^2+^ (HBSS-/-; Chemical reagents, Beijing, China). The purity of the neutrophils was above 95%. Neutrophils were then suspended in HBSS with Ca^2+^/Mg^2+^ (HBSS+/+; Chemical reagents) to a concentration of 2.5 × 10^7^ cells/ml and used for ANCA antigen translocation analysis, respiratory burst measurements, neutrophil degranulation and detection of S1P in neutrophil supernatant [35]. This research was in compliance with the Declaration of Helsinki and approved by the clinical research ethics committee of the Peking University First Hospital. Written informed consent was obtained from each participant.

### Inhibition of the S1P receptor

Previous studies have showed that neutrophils express S1PR1, 4 and 5 [36]. VPC23019 (Tocris, Louis, USA) is a specific antagonist for S1P receptor 1 and 3 [37]. CYM50358 (Tocris) is a specific antagonist for S1P receptor 4 [38]. FTY720 is a structural analog of sphingosine, as well as being phosphorylated by Sphk, which then acts with four S1P receptors (S1P_1_, S1P_3_, S1P_4_ and S1P_5_) [28]. FTY720 (Selleck, Houston, USA) could abolish the biological effect of S1P and cover all types of S1P receptors on neutrophils. The concentration of the above-mentioned S1P receptor antagonists was first investigated. Cells were incubated with VPC23019 or CYM50358 at different doses (25nM; 50nM; 100nM; 200nM) for 15 minutes. VPC23019 and CYM50358 inhibited S1P-primed neutrophils for ANCA-induced activation at 100nM for 15 minutes. The highest inhibition rates of VPC23019 and CYM50358 were 26% and 27%, respectively. The highest inhibition rate of FTY720 (50nM, 15 minutes) in S1P-primed neutrophils for ANCA-induced activation was 68%.

In C5a-primed neutrophils for ANCA-induced activation, cells were incubated with FTY720 for different doses and time points (10nM, 5 minutes; 50nM, 5 minutes; 100nM, 5 minutes; 10nM, 15 minutes; 50nM, 15 minutes; 100nM, 15 minutes). We selected FTY720 (50nM) at 15 minutes for the experiments because we found the inhibition rate (83%) was highest. The toxicity of FTY720 to neutrophils had been examined by fluorescence-activated cell sorting (FACS) using a Cell Apoptosis Detection Kit (BD Biosciences, CA, USA). Pre-incubated with FTY720, the proportion of living cells was higher than 90%. Cells were pre-incubated with 50nM FTY720 or its vehicle, dimethyl sulfoxide (DMSO) as the control, followed by other treatments. The inhibition rate was calculated according to the following formula:

$$\begin{array}{ll} \text{Inhibition}\;\text{rate}\;=\; & \left({\mathrm{MFI}}_{\mathrm{DMSO}+C5a+\mathrm{ANCA}}-{\mathrm{MFI}}_{\mathrm{FTY}720+C5a+\mathrm{ANCA}}\right)\\ & /\left({\mathrm{MFI}}_{\mathrm{DMSO}+C5a+\mathrm{ANCA}}-{\mathrm{MFI}}_{\mathrm{DMSO}}\right)\;\times \;100\% \end{array}$$

where MFI is the mean fluorescence intensity.

### Membrane expression of PR3 and MPO on neutrophils after priming

Flow cytometry was used to evaluate PR3 and MPO expression on neutrophils. Cells were incubated with C5a (100 ng/ml) (Biovision, San Francisco, CA, USA) or buffer control for 45 minutes at 37°C. For S1P priming groups, cells were incubated with S1P (100, 500 and 1000 nmol/L) (Sigma-Aldrich, Louis, USA) or buffer control for 30 minutes at 37°C. Doses for S1P stimulation were all above the dissociation constant for the receptors. All further steps were performed on ice and washing steps were carried out using HBSS +/+containing 1% bovine serum albumin (BSA). Neutrophils were incubated with 0.5 mg/ml heat-aggregated goat IgG for 15 minutes to saturate Fcγ receptors. Next, cells were stained with a saturating dose of mouse monoclonal IgG1 antibody directed against human PR3 (Clone number: WGM2) or MPO (Clone number: 2C7) (Abcam, Cambridge, UK) or with an irrelevant IgG1 control antibody (Biolegend, CA, USA) for 30 minutes. Neutrophils were then incubated with phycoerythrin (PE)-conjugated goat anti-mouse antibody (Abcam, Cambridge, UK) in the presence of 0.5 mg/ml heat-aggregated goat IgG. Fluorescence intensity of PE was analyzed using flow cytometry assessment of ANCA-antigen expression. Samples were analyzed using the FACScan (Becton Dickinson, Heidelberg, Germany). Neutrophils were identified in the scatter diagram, and data were collected from 10,000 cells per sample. The level of PR3- or MPO-expression was calculated as the MFI of specific binding of the isotype control antibody. For the inhibition test, cells were pre-incubated with FTY720 (50nM) or its vehicle, as control, followed by other treatments [35].

### Detection of MPO in neutrophil supernatant by ELISA

MPO in the S1P or C5a-primed neutrophil supernatant was tested by ELISA using a commercial kit (USCNK, Wuhan, China). Cells were incubated with S1P (100, 500 and 1000 nmol/L) (Sigma-Aldrich) or buffer control for 30 minutes at 37°C. Supernatant fluids were collected and used for ELISA analysis. In brief, the microtiter plate provided in this kit has been pre-coated with an antibody specific to MPO. The cloaked antibody was a monoclonal antibody in this assay. The detection antibody was a polyclonal antibody. Supernatants of neutrophils at dilutions of 1:200 and standards were then added to the appropriate microtiter plate-wells with a biotin-conjugated antibody preparation specific for MPO. Next, Avidin conjugated to horseradish peroxidase (HRP) was added to each microplate well and incubated. After 3,3′-5,5′ tetramethylbenzidin (TMB) substrate solution was added, only those wells that contained MPO, biotin-conjugated antibody and enzyme-conjugated Avidin would exhibit a change in color. The enzyme-substrate reaction was terminated by the addition of sulphuric acid solution and the color change is measured spectrophotometrically at a wavelength of 450 nm. The concentration of MPO in the samples was then determined by comparing the optical density (OD) of the samples to the standard curve.

### Detection of S1P by ELISA

S1P was tested by ELISA using a commercial kit (Echelon,Utah, USA). The 96-well microtiter plate was coated with S1P and blocked to reduce non-specific binding. Then we mixed the S1P standard and samples with the anti-S1P antibody before adding the mixture to the S1P-coated plate. The antibody competes for binding to S1P bound to the plate or in the sample. Following an incubation and plate wash, streptavidin-HRP was added to the plate and bound to all anti-S1P antibodies (labeled with biotin) bound to the plate. After an additional incubation and plate wash, TMB substrate was added to the plate and the reaction stopped by the addition of sulfuric acid. The absorbance at 450 nm was measured. The concentration of S1P in the samples was determined by comparison to the standard curve [39].

### Measurement of respiratory burst by oxidation of dihydrorhodamine (DHR) to rhodamine

The generation of reactive oxygen radicals was assessed using DHR. This method was based on the fact that reactive oxygen radicals cause an oxidation of the non-fluorescence DHR to the green fluorescence rhodamine. Isolated neutrophils were gradually warmed to 37°C and incubated with 0.05 mM DHR123 (Sigma-Aldrich) for 10 minutes at 37°C. Sodium azide (NaN_3_) (2 mM) was added in order to prevent intracellular breakdown of H_2_O_2_ by catalase. Then, neutrophils were primed with S1P for 30 minutes or C5a for 45 minutes at 37°C and incubated with patient-derived ANCA-positive IgG (200 μg/ml), normal IgG for 1 h at 37°C. The reaction was stopped by addition of 1 ml of ice-cold HBSS/1% BSA. Samples were kept on ice and analyzed using a FACScan. Neutrophils were identified in the scatter diagram, and data were collected from 10,000 cells per sample. The shift of green fluorescence in the FL-1 mode was determined. For each condition, the MFI (representing the amount of generated reactive oxygen radicals) was reported [34,35].

### ANCA-activated S1P or C5a-primed neutrophils degranulation

Lactoferrin, an iron binding multifunctional glycoprotein that was an abundant component of the specific granules of neutrophils, was considered as a biomarker of neutrophil degranulation [40-42]. Neutrophils were stimulated with S1P or C5a followed by stimulation with MPO-ANCA-positive IgG or PR3-ANCA-positive IgG, normal IgG or buffer control for 1 h, respectively. Cells were pre-incubated with the S1P receptor antagonist or its vehicle, DMSO, as control for 15 minutes on ice before the priming. Lactoferrin in the neutrophil supernatant was tested by ELISA using a commercial kit (USCNK) as a measure of neutrophil degranulation. The ELISA procedure of measuring lactoferrin was as described previously [43]. The concentrations of lactoferrin in the samples were then determined by comparing the OD value of the samples to the standard curve.

### Membrane expression of CD88 on neutrophils

Flow cytometry was used to evaluate CD88 expression on neutrophils. Cells were incubated with S1P (500 nmol/L) (Sigma-Aldrich), supernatant of C5a- stimulated neutrophils or buffer control for 30 minutes. All further steps were carried out using HBSS +/+containing 1% BSA. Next, cells were stained with a saturating dose of PE-conjugated mouse monoclonal IgG1 antibody directed against human CD88 (Biolegend, CA, USA) or with an irrelevant IgG1 control antibody (Biolegend, California, USA) for 30 minutes. Fluorescence intensity of PE was analyzed using flow cytometry assessment of CD88 expression. Samples were analyzed using a FACScan (Becton Dickinson, Heidelberg, Germany). Neutrophils were identified in the scatter diagram, and data were collected from 10,000 cells per sample. The level of CD88 expression was calculated as MFI of specific binding of the isotype control antibody.

### Neutrophil migration

To test the effect of S1P receptor antagonist on the C5a-induced neutrophil migration, neutrophils were preincubated with 50nM S1P receptor antagonist or vehicle control for 15 minutes: 4 × 10^5^ cells were loaded in the upper chamber of a Transwell insert (Corning, NY, USA) with 3.0-μm pores in 12-well plates. C5a (100 ng/ml) was placed in the lower chamber. The plates were then incubated at 37°C with 5% CO_2_ for 90 minutes. Neutrophils without treatment of S1P receptor antagonist in the upper chamber or wells without C5a in the lower chamber were used as the controls. The number of neutrophils that migrated across the filter was counted using FACS [44].

### Statistical analysis

The Shapiro-Wilk test was used to examine whether the data were normally distributed. Quantitative data were expressed as means ± SD (for data that were normally distributed) or median and range (for data that were not normally distributed). Differences in quantitative parameters between groups were assessed using the *t*-test (for data that were normally distributed) or Mann-Whitney *U*-test (for data that were not normally distributed) as appropriate. Differences were considered significant at *P* <0.05. Analysis was performed with SPSS statistical software package (version 16.0, Chicago, IL, USA).

## Results

### Plasma levels of S1P were elevated in AAV patients in the active stage compared with remission

**Figure 1:**
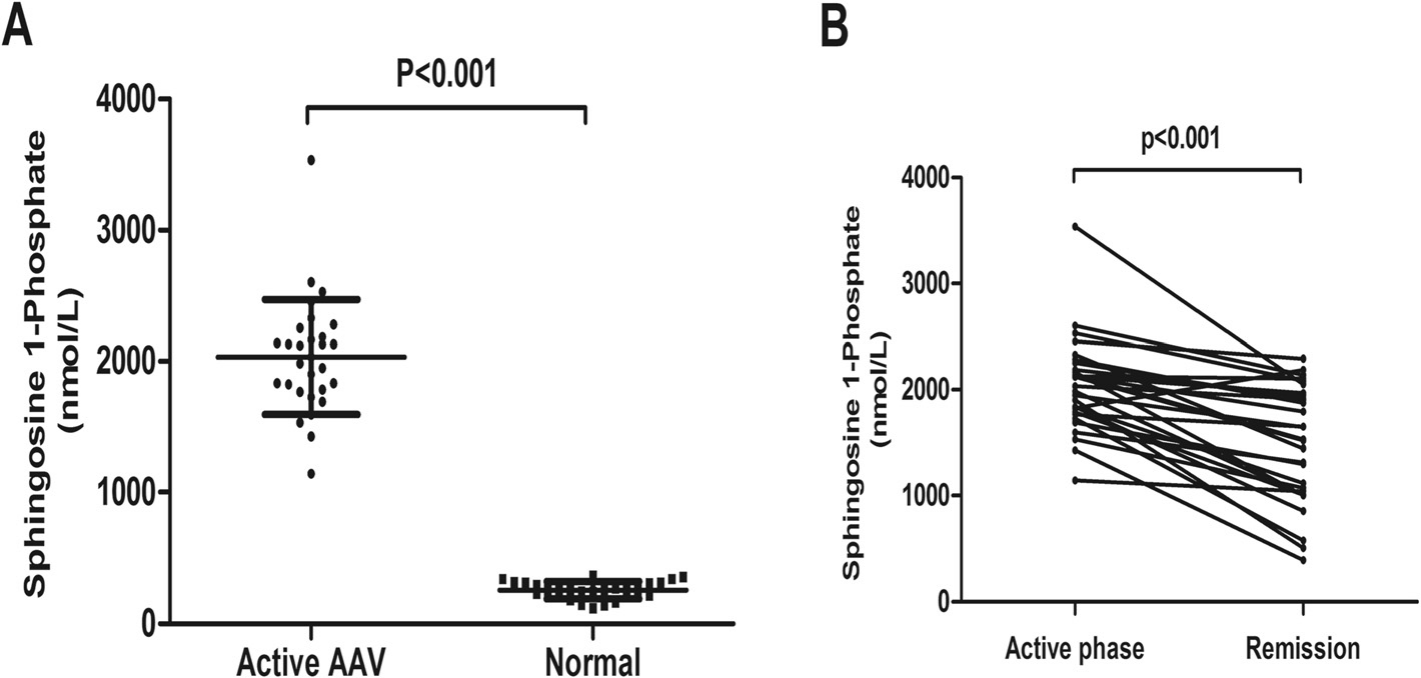
**Plasma levels of sphingosine-1-phosphate (S1P) in patients with antineutrophil cytoplasmic antibody (ANCA)-associated vasculitis (AAV) at the active stage and in remission. (A)** Plasma levels of S1P in AAV patients compared with normal controls. **(B)** Plasma levels of S1P in AAV patients at active stage and in remission.

Among the 29 patients with AAV, 14 (48.3%) were male and 15 (51.7%) were female, with an age of 58.5 ± 12.8 years at diagnosis. Two patients were cytoplasmic ANCA (cANCA)-positive and all these sera recognized PR3; 27 patients were perinuclear ANCA (pANCA)-positive and all these sera recognized MPO. The level of initial serum creatinine was 312.7 ± 237.6 (range 62.0 to 1301.0) μmol/L. The levels of BVAS in the 29 patients were 22.3 ± 5.9 in the active stage, and all were zero in remission. The plasma levels of circulating S1P were significantly higher in AAV patients with active disease compared with AAV patients in remission and normal controls (2034.2 ± 438.5 versus 1489.3 ± 547.4 nmol/L, *P* <0.001; 2034.2 ± 438.5 versus 254.3 ± 69.9 nmol/L, *P* <0.001, respectively). Except for only one patient with AAV, the plasma level of S1P in the active stage was higher than that in remission for each AAV patient (Figure 1).

### S1P increased translocation of ANCA antigens

**Figure 2:**
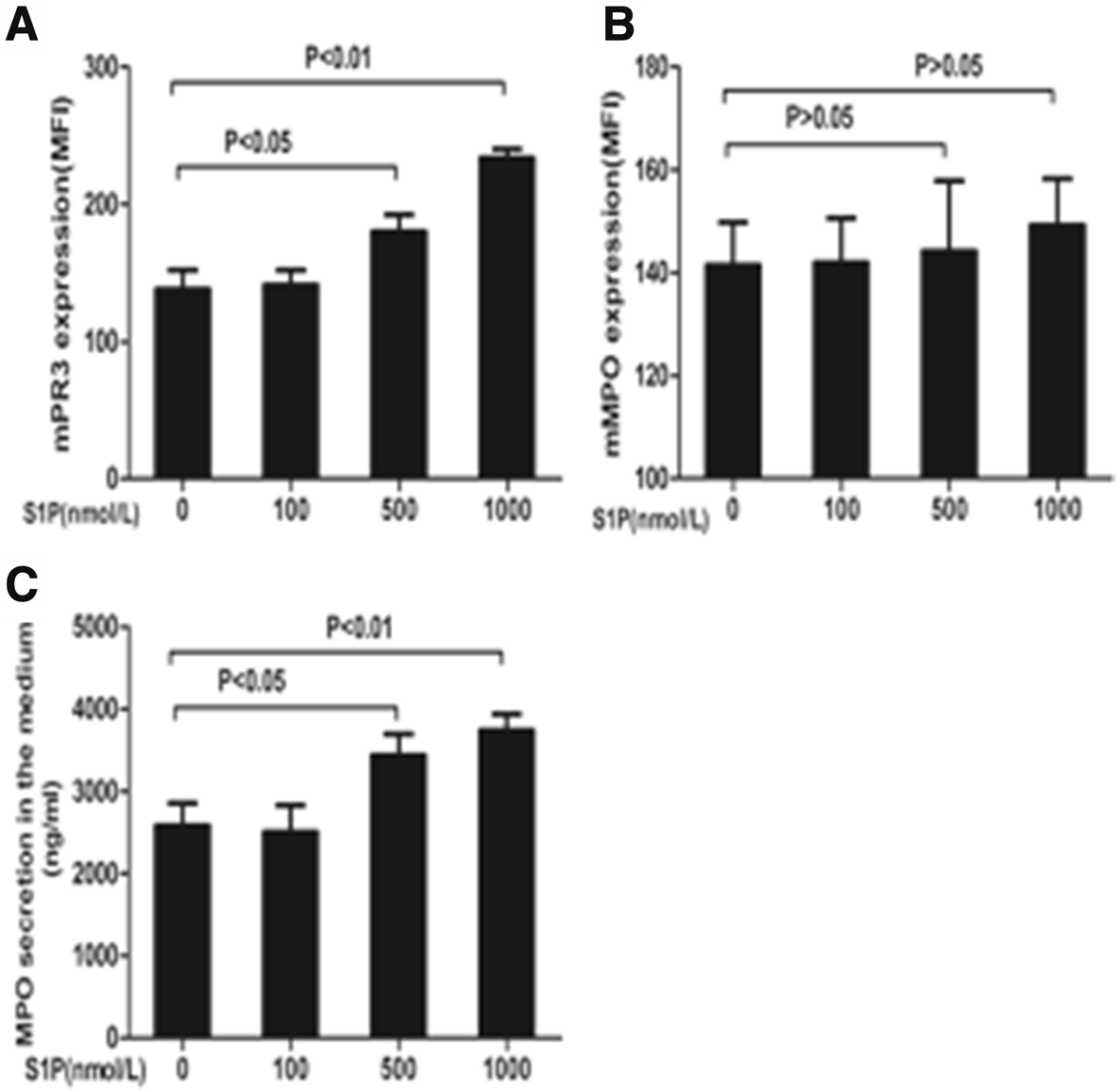
**Sphingosine-1-phosphate (S1P) increased translocation of antineutrophil cytoplasmic antibody (ANCA) antigens on neutrophils.** Human neutrophils were isolated and incubated with different concentrations of S1P for 30 minutes. mPR3 and myeloperoxidase (MPO) were measured with flow cytometry or by ELISA and compared with non-stimulated cells. **(A)** mPR3 expression compared with non-primed nertrophils. **(B)** mMPO expression compared with non-primed nertrophils. **(C)** MPO concentration compared with non-primed neutrophils. Bars represent mean ± SD of repeated measurements of neutrophils from five independent experiments and donors.

Expression of mPR3 on neutrophils of eight healthy blood donors was analyzed. Neutrophils were incubated with different concentrations of S1P (100, 500 and 1000 nmol/L), and mPR3 expression was determined by flow cytometry. The level of mPR3 expression on neutrophils increased (mPR3 expression on neutrophils were 138.0 ± 13.9, 141.0 ± 11.1, 180.0 ± 12.4, 233.8 ± 6.6 for 0, 100, 500 and 1000 nmol/L S1P, respectively, expressed as MFI). Compared with non-primed neutrophils, the level of mPR3 expression was significantly higher on neutrophils primed with S1P at concentrations of 500 and 1000 nmol/L (*P* <0.05; *P* <0.01), respectively (Figure 2A). mMPO expression on neutrophils was 141.5 ± 8.3, 142.0 ± 8.6, 144.3 ± 13.6, 149.3 ± 9.1 for 0, 100, 500 and 1,000 nmol/L S1P, respectively, expressed as MFI). Increases in membrane-bound PR3 expression were much stronger during neutrophils priming compared with mMPO (Figure 2B). Neutrophils were incubated with different concentrations of S1P (100, 500 and 1000 nmol/L), and MPO concentration in the S1P-primed neutrophil supernatant was detected by ELISA. The concentration of MPO in the S1P-primed neutrophil supernatant increased (the MPO concentrations were 2579.3 ± 278.3, 2507.0 ± 325.2, 3436.0 ± 258.7, 3739.7 ± 194.7, for 0, 100, 500 and 1,000 nmol/L S1P, respectively). Compared with non-primed neutrophils, the concentration of MPO was significantly higher in the neutrophil supernatant primed with S1P at concentrations of 500 and 1,000 nmol/L (*P* <0.05; *P* <0.01), respectively (Figure 2C).

### S1P-primed neutrophils for ANCA-induced respiratory burst and degranulation

**Figure 3:**
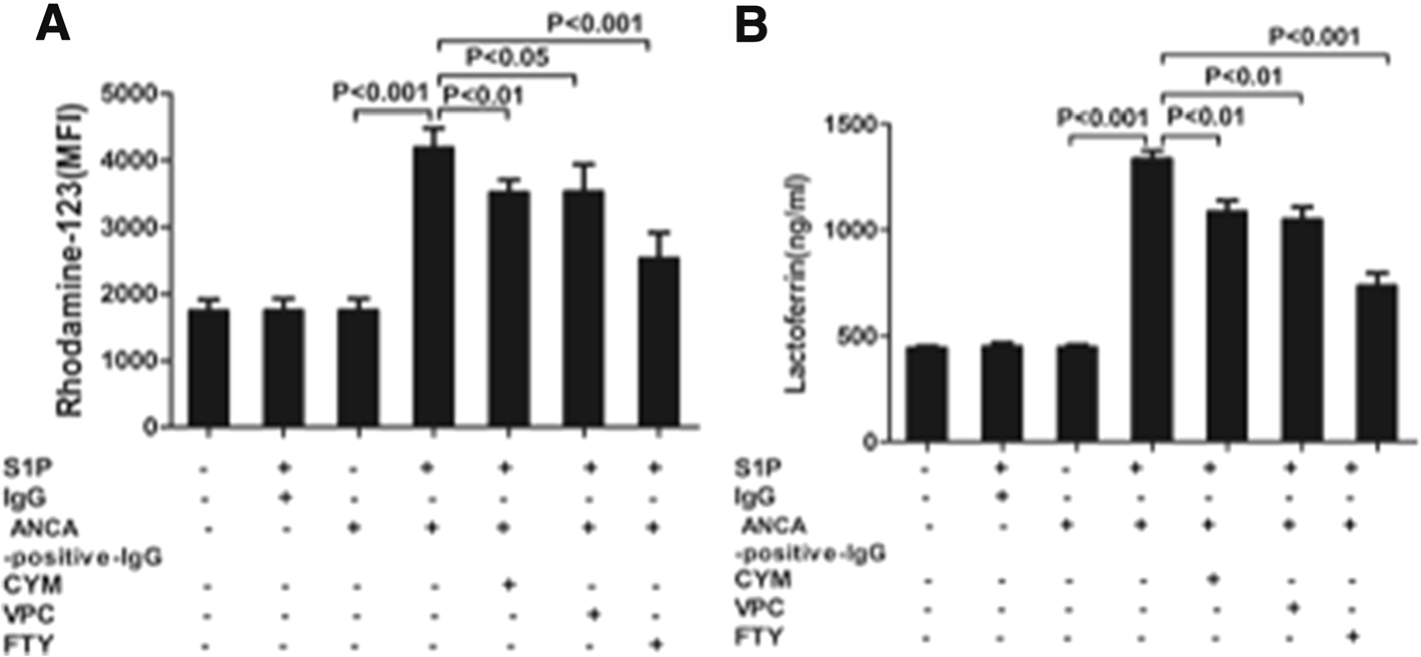
**Sphingosine-1-phosphate (S1P)-primed neutrophils for antineutrophil cytoplasmic antibody (ANCA)-induced respiratory burst and degranulation. (A)** Neutrophil respiratory burst induced by patient-derived ANCA-positive IgG was measured by conversion of dihydrorhodamine-123 (DHR-123) to rhodamine-123 in S1P-primed cells in the presence and absence of CYM50358, VPC23019 and FTY720, respectively. **(B)** ANCA-induced neutrophil degranulation was determined by measuring the lactoferrin concentrations in the supernatant of neutrophil degranulation reaction. CYM50358, VPC23019 and FTY720 reduced ANCA-induced lactoferrin release.

We studied whether S1P primed neutrophils for ANCA-induced respiratory burst and degranulation. ANCA-postive IgG was prepared from three patients with active MPO-ANCA-positive vasculitis and two patients with active PR3-ANCA-positive vasculitis, respectively. Based on the observation described above that S1P at a concentration of 500 nmol/L significantly increased mPR3 expression on neutrophils, this concentration of S1P was employed for testing ANCA-induced respiratory burst and degranulation. Compared with non-primed neutrophils, the MFI value for DHR oxidation increased significantly in S1P-primed neutrophils activated with ANCA-positive IgG (1,735.0 ± 173.7 versus 4,190.5 ± 294.6, *P* <0.001; and decreased to 3,514.8 ± 194.4 (*P* <0.01), 3,533.0 ± 408.3 (*P* <0.05) and 2,524.5 ± 392.4 (*P* <0.001) pre-incubation with CYM50358, VPC23019 and FTY720, respectively) (Figure 3A). The highest inhibition rates of VPC23019, CYM50358 and FTY720 were 26%, 27%, and 68%, respectively. No obvious respiratory burst activity was observed with S1P alone. ANCA-induced neutrophil degranulation was determined by measuring the lactoferrin concentration in the supernatant. In S1P-primed neutrophils induced by ANCA-positive IgG, the lactoferrin concentration in the supernatant increased from 443.0 ± 9.0 ng/ml in untreated cells to 1,366.0 ± 30.3 ng/ml (*P* <0.001) and decreased to 1,085.3 ± 53.7 (*P* <0.01), 1,048.3 ± 60.2 (*P* <0.01) and 734.3 ± 61.9 (*P* <0.001) pre-incubation with CYM50358, VPC23019 and FTY720, respectively (Figure 3B).

### S1P receptor antagonist inhibited C5a-primed neutrophils for ANCA-induced respiratory burst and degranulation

**Figure 4:**
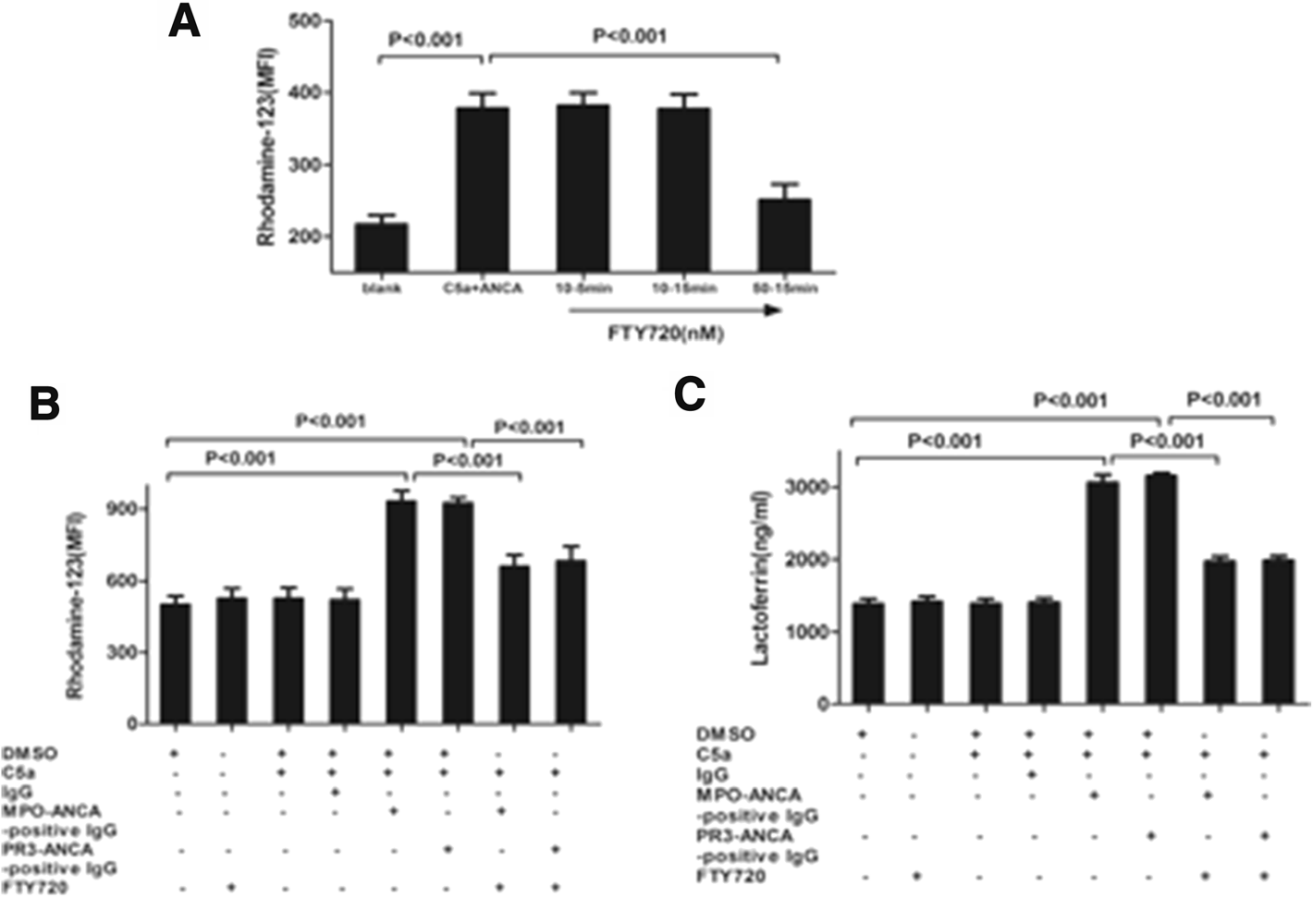
**Sphingosine-1-phosphate (S1P) receptor antagonist inhibited C5a-primed neutrophils for antineutrophil cytoplasmic antibody (ANCA)-induced respiratory burst and degranulation. (A)** Neutrophils were incubated with different concentrations of FTY720, and the mean fluorescence intensity (MFI) value for dihydrorhodamine (DHR) oxidation decreased, compared with C5a-primed neutrophils for ANCA-induced activation. Cells were incubated with FTY720 at different concentrations and times (10nM, 5 minutes; 10nM, 15 minutes; 50nM, 15 minutes; respectively). **(B)** Neutrophil respiratory burst induced by patient-derived myeloperoxidase (MPO)-ANCA-positive IgG or proteinase-3 (PR3)-ANCA-positive IgG was measured by conversion of DHR-123 to rhodamine-123 in C5a-primed cells. Inhibition of S1P reduced C5a-primed neutrophils for ANCA-induced respiratory burst. **(C)** ANCA-induced neutrophil degranulation was determined by measuring the lactoferrin concentrations in the supernatant of neutrophil degranulation reaction. Inhibition of S1P reduced ANCA-induced lactoferrin release. Bars represent mean ± SD of repeated measurements of neutrophils from five independent experiments and donors.

Based on our previous study [45] that C5a at a concentration of 100 ng/ml significantly increased C5a-primed neutrophils for ANCA-induced respiratory burst and degranulation, this concentration of C5a was employed for this inhibition experiment. Neutrophils were incubated with different concentrations of FTY720, and the MFI value for DHR oxidation decreased compared with C5a-primed neutrophils for ANCA-induced activation (Figure 4A). Compared with non-primed neutrophils, the MFI value for DHR oxidation increased significantly in C5a-primed neutrophils activated with MPO-ANCA-positive IgG and PR3-ANCA-positive IgG (932.5 ± 43.5 versus 501.0 ± 36.4, *P* <0.001; 925.0 ± 23.9 versus 501.0 ± 36.4, *P* <0.001, respectively). Neutrophils were pre-incubated with the S1P receptor antagonist before the priming with C5a and the subsequent stimulation with ANCA. Pre-incubation of neutrophils with the S1P receptor antagonist decreased oxygen radical production in C5a-primed neutrophils induced by ANCA-positive IgG from patients. Pre-incubation of neutrophils with the S1P receptor antagonist did not significantly decrease mPR3 expression and MPO release in the C5a-primed neutrophil supernatant (data not shown). In C5a-primed neutrophils, subsequently activating with MPO-ANCA-positive IgG, the MFI value for DHR oxidation was 932.5 ± 43.5, which decreased to 659.8 ± 48.4 upon pre-incubation with S1P receptor antagonist (compared with that without the antagonist, *P* <0.001, the inhibition rate was 63.1 ± 3.3%). For PR3-ANCA-positive IgG, the MFI value for DHR oxidation was 925.0 ± 23.9 in C5a-primed neutrophils, which decreased to 681.3 ± 61.6 upon pre-incubation with S1P receptor antagonist (compared with that without the antagonist, *P* <0.001, the inhibition rate was 57.3 ± 7.1%) (Figure 4B).

Pretreatment with S1P receptor antagonist significantly reduced MPO-ANCA-positive IgG-induced and PR3-ANCA-positive IgG-induced lactoferrin release. The lactoferrin concentration increased from 1,387.5 ± 71.2 ng/ml in the non-primed neutrophil supernatant to 3,059.8 ± 109.0 ng/ml in C5a-primed neutrophils induced by MPO-ANCA-positive IgG supernatant (*P* <0.001), and decreased to 1,966.3 ± 72.3 ng/ml upon pre-incubation with S1P receptor antagonist (compared with that without the antagonist, *P <*0.001, the inhibition rate was 65.2 ± 3.1%). In C5a-primed neutrophils induced by PR3-ANCA-positive IgG, the lactoferrin concentration in the supernatant increased from 1,387.5 ± 71.2 ng/ml in untreated cells to 3,150.3 ± 41.9 ng/ml (*P* <0.001), which decreased to 1,982.3 ± 64.7 ng/ml upon pre-incubation with S1P receptor antagonist (compared with that without the antagonist, *P* <0.001, the inhibition rate was 66.3 ± 5.4%) (Figure 4C).

### Supernatant of C5a-stimulated neutrophils primed fresh neutrophils for ANCA-mediated respiratory burst and degranulation

**Figure 5:**
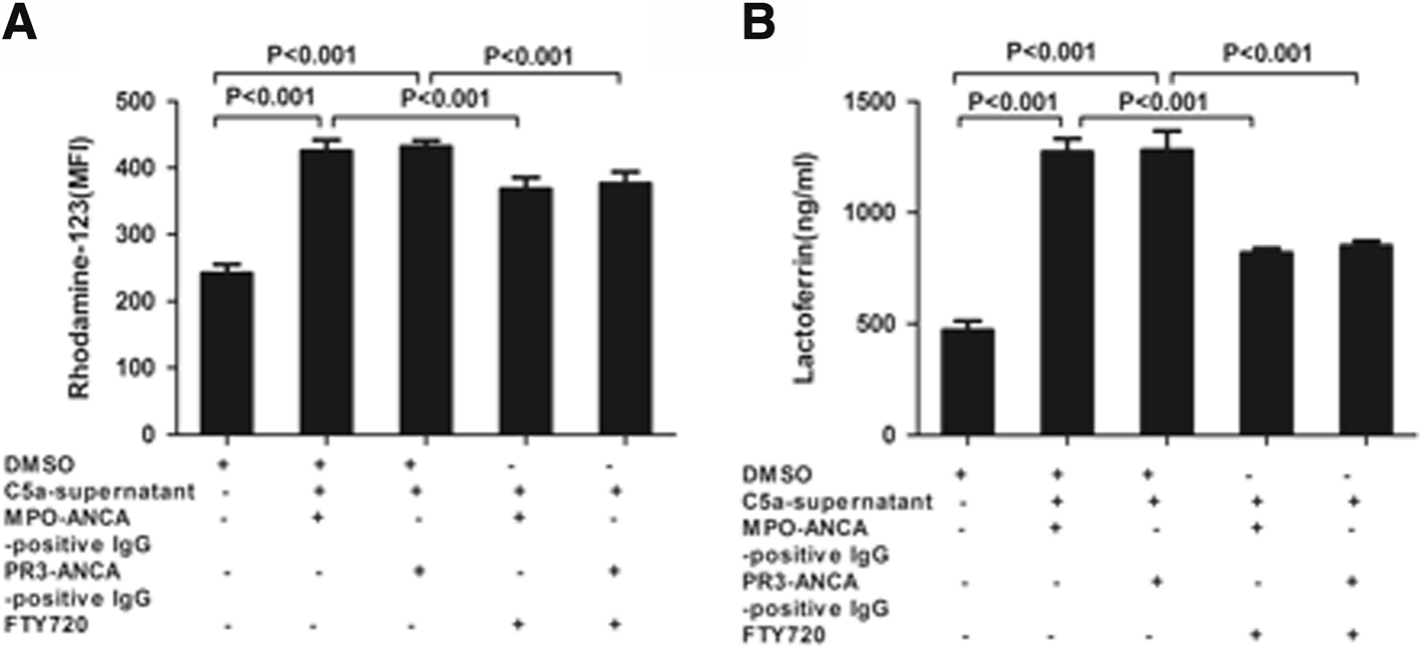
**Sphingosine-1-phosphate (S1P) receptor antagonist inhibited supernatant (C5a-stimulated neutrophils) -primed neutrophils for antineutrophil cytoplasmic antibody (ANCA)-induced respiratory burst and degranulation. (A)** Neutrophil respiratory burst induced by patient-derived MPO-ANCA-positive IgG or PR3-ANCA-positive IgG was measured by conversion of dihydrorhodamine-123 (DHR-123) to rhodamine-123 in supernatant-primed cells. Inhibition of S1P reduced supernatant (C5a-stimulated neutrophils)-primed neutrophils for ANCA-induced respiratory burst. **(B)** Inhibition of S1P reduced supernatant (C5a-stimulated neutrophils)-primed neutrophils for ANCA-induced lactoferrin release. Bars represent mean ± SD of repeated measurements of neutrophils from five independent experiments and donors.

Neutrophils were incubated with C5a-stimulated neutrophils supernatant and the effect was inhibited by the S1P receptor antagonist. Compared with non-primed neutrophils, the MFI value for DHR oxidation increased significantly in supernatant-primed neutrophils activated with MPO-ANCA-positive IgG and PR3-ANCA-positive IgG (425.2 ± 16.6 versus 242.2 ± 13.0, *P* <0.001; 432.0 ± 8.9 versus 242.2 ± 13.0, *P* <0.001, respectively). Neutrophils were pre-incubated with the S1P receptor antagonist, that is, FTY720, before priming with supernatant and the subsequent stimulation with ANCA. Pre-incubation of neutrophils with the S1P receptor antagonist decreased oxygen radical production in C5a primed neutrophils induced by ANCA-positive IgG from patients. In supernatant-primed neutrophils, subsequently activating with MPO-ANCA-positive IgG, the MFI value for DHR oxidation was 425.2 ± 16.6, which decreased to 368.2 ± 17.3 upon pre-incubation with S1P receptor antagonist (compared with that without the antagonist, *P* <0.001). For PR3-ANCA-positive IgG, the MFI value for DHR oxidation was 432.0 ± 8.9 in supernatant-primed neutrophils, which decreased to 376.2 ± 18.2 upon pre-incubation with S1P receptor antagonist (compared with that without the antagonist, *P* <0.001) (Figure 5A).

Pretreatment with S1P receptor antagonist significantly reduced MPO-ANCA-positive IgG-induced and PR3-ANCA-positive IgG-induced lactoferrin release. The lactoferrin concentration increased from 427.6 ± 104.0 ng/ml in the non-primed neutrophils supernatant to 1,105.0 ± 380.1 ng/ml in supernatant-primed neutrophils induced by MPO-ANCA-positive IgG supernatant (*P* <0.001), and decreased to 731.0 ± 201.9 ng/ml upon pre-incubation with S1P receptor antagonist (compared with that without the antagonist, *P <*0.001). In supernatant-primed neutrophils induced by PR3-ANCA-positive IgG, the lactoferrin concentration increased from 427.6 ± 104.0 ng/ml in untreated cells to 1,110.2 ± 389.2 ng/ml (*P* <0.001), which decreased to 750.4 ± 224.1 ng/ml upon pre-incubation with S1P receptor antagonist (compared with that without the antagonist, *P* <0.001) (Figure 5B).

### S1P or supernatant of C5a-stimulated neutrophils upregulated CD88 expression on neutrophils

**Figure 6:**
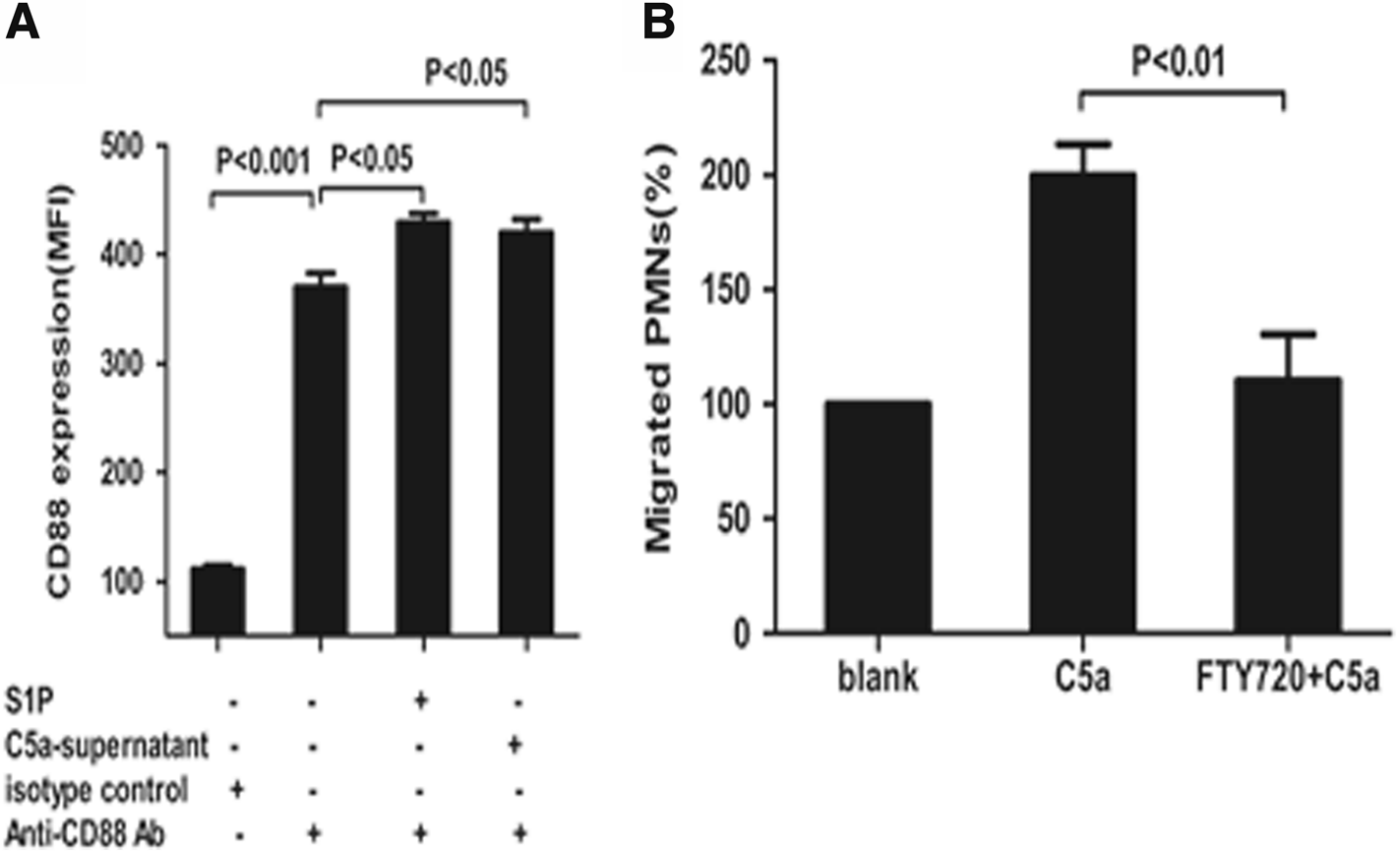
**Sphingosine-1-phosphate (S1P) or supernatant of C5a-stimulated neutrophils upregulated CD88 expression on neutrophils and S1P receptor antagonist reduced C5a-induced neutrophil migration. (A)** CD88 expression increased on neutrophils after S1P or supernatant of C5a-stimulated neutrophil engagement in neutrophils. Neutrophils were stimulated with S1P 500 nmol/L, supernatant of C5a-stimulated neutrophils or buffer control for 30 minutes. The expression of CD88 was determined by FACS. A representative example of five independent experiments is shown. **(B)** Isolated neutrophils were pre-incubated with S1P receptor antagonist at 50 nM and loaded into the upper chamber. After the upper chamber neutrophil co-incubation with C5a 100 ng/ml in the lower chamber, the migration was assayed through counting the number of migrated neutrophils using FACS. Results are presented as the percentage of migrated neutrophils after C5a induced or pre-incubated with S1P receptor antagonist compared with the blank. A representative example of three independent experiments is shown.

CD88 expression on neutrophils increased after S1P engagement. Compared with non-triggered neutrophils, the MFI values for CD88 expression were significantly higher than in S1P-triggered neutrophils (369.8 ± 29.1 versus 429.4 ± 18.5, *P* <0.05). The MFI values for CD88 expression increased from 369.8 ± 29.1 in the non-triggered neutrophils to 420.0 ± 27.5 in the supernatant-primed neutrophils (*P* <0.05) (Figure 6A).

### S1P receptor antagonist inhibited C5a-induced neutrophil migration

To clarify the involvement of S1P on C5a-induced neutrophil migration, we investigated the effect of S1P receptor antagonist on neutrophil migration. Pre-incubation with S1P receptor antagonist significantly inhibited C5a-induced neutrophil migration (the number of migrated neutrophils was 110.5 ± 20.0 versus 200.0 ± 13.5, *P* <0.01) (Figure 6B).

## Discussion

ANCA-induced neutrophil respiratory burst and degranulation are important contributors to the development of AAV. Recent studies, both in the mouse model and in humans, suggest that complement activation is involved in the pathogenesis of AAV [10-15,45-47]. In particular, the interaction between C5a and C5aR (CD88) plays a central role in ANCA-mediated neutrophil recruitment and activation [15]. Our previous study confirmed this observation by demonstrating that purified recombinant C5a increased ANCA antigen translocation and ANCA-mediated respiratory burst in C5a-stimulated neutrophils [45]. However, the intracellular molecular mechanism triggered by C5a in neutrophils has not been fully identified.

It is becoming more evident that certain classes of membrane lipids can be modified in a regulated manner to generate bioactive lipid second messengers. S1P is generated by the conversion of ceramide to sphingosine by the enzyme ceramidase and the subsequent conversion of sphingosine to S1P, which is a potent bioactive sphingolipid metabolite that regulates inflammation and immune responses [48].

Several studies have reported that sphingosine acts as an endogenous regulator of neutrophil functions [23,49,50]. It was reported that low concentration of S1P promotes inflammatory cell chemotaxis [51,52]. Furthermore, it has been proposed that Sphk regulates neutrophil priming to provide an essential defense against infections [50], and to mediate neutrophil inflammatory responses [23,50]. The current study found that the level of circulating S1P was significantly higher in AAV patients with active disease compared with those in remission. More importantly, S1P was found to prime neutrophils for ANCA-induced respiratory burst and degranulation. Therefore, we speculated that S1P might promote the development of inflammation and disease activity of AAV.

In our study, it was found that S1P receptor antagonist downregulated C5a-induced neutrophil migration and significantly attenuated C5a-primed neutrophils for ANCA-induced respiratory burst and degranulation, with an inhibition rate of about 80%, which suggests that the S1P played an important role in C5a-primed neutrophils for ANCA-mediated activation. In addition, S1P upregulated CD88 expression on neutrophils, which suggests that there may be an S1P-C5a loop in ANCA-induced neutrophil activation. It was reported that S1P is involved in several immune responses of C5a, which C5a rapidly stimulates the generation of S1P, Sphk1 activity, and membrane translocation of Sphk1 in human monocyte-derived macrophages [53-56]. Bachmaier *et al*. reported that Sphk1 regulates the balance between expression of CD88 and C5L2 on phagocytes in experimental lung inflammatory injury [30].

In the previous study, it was found that reactive oxygen species increased in TNFα-primed human monocytes for ANCA-induced activation [57]. Presumably, S1P might induce a similar effect on monocytes, which is of special interest for further investigation.

## Conclusions

**Figure 7:**
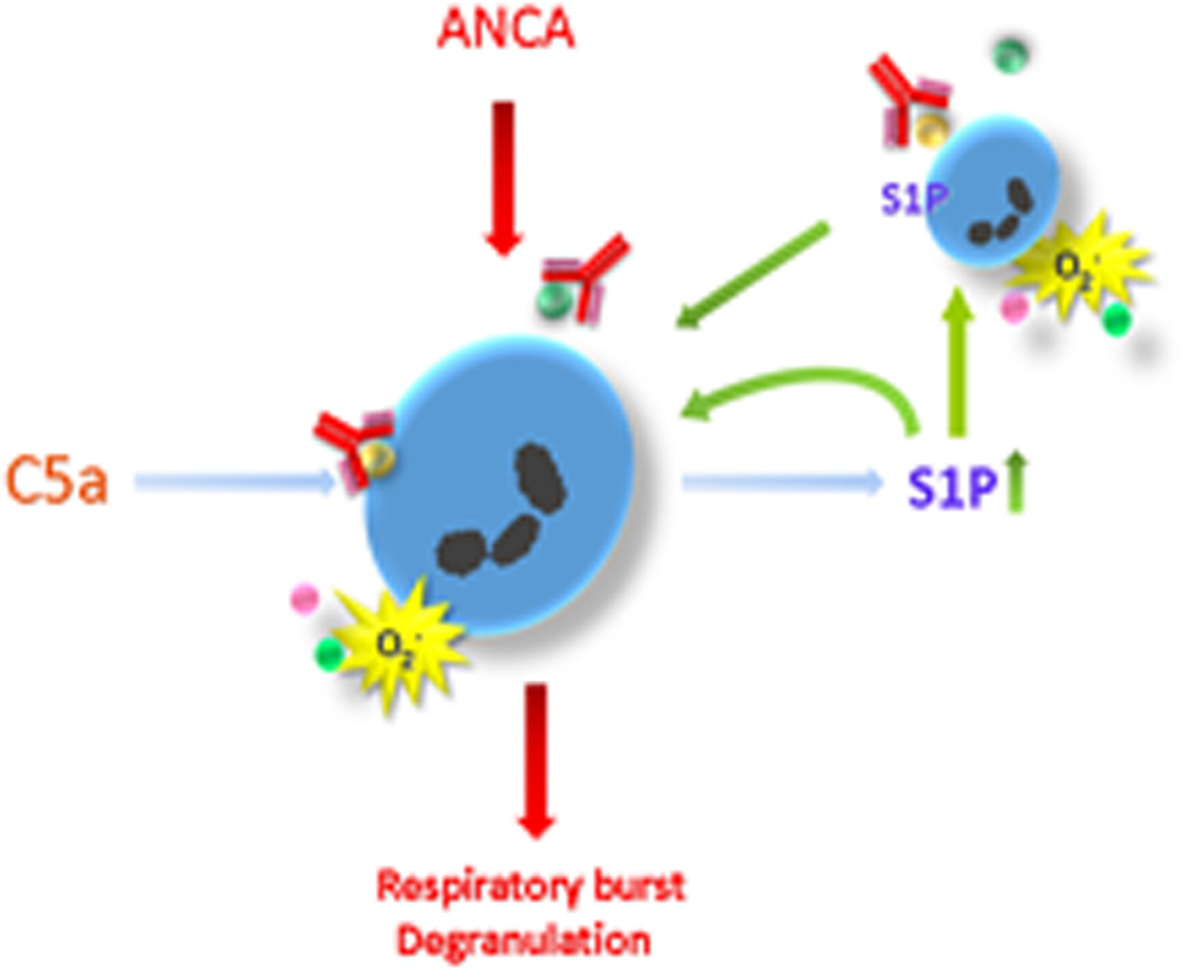
**Proposed working model for the interaction between C5a and sphingosine-1-phosphate (S1P) in antineutrophil cytoplasmic antibody (ANCA)-mediated neutrophils activation.** Neutrophils are primed by C5a to express ANCA antigens at the cell surface and supernatant. ANCA antibodies interact with the ANCA antigens which result in primed-neutrophil activation. The ANCA-activated neutrophils release S1P that can further activate neutrophils. S1P receptor antagonist may attenuate C5a-primed neutrophils for ANCA induced activation.

S1P triggered by C5a-primed neutrophils could further activate neutrophils. Blocking S1P may attenuate C5a-induced activation of neutrophils by ANCA (Figure 7). The interaction between S1P and C5a plays an important role in neutrophils for ANCA-mediated activation. The findings presented in this study opened a new aspect to better understand the intracellular signaling cascades triggered by C5a and indicated a potential therapeutic candidate for controlling inflammatory injury in AAV.

## Abbreviations

AAV: antineutrophil cytoplasmic antibody-associated vasculitis; ANCA: antineutrophil cytoplasmic antibody; BSA: bovine serum albumin; BVAS: Birmingham vasculitis activity score; C5aR: C5a receptor; C5L2: C5a receptor-like 2; DHR: dihydrorhodamine; EDTA: ethylene diamine tetraacetic acid; EGPA: eosinophilic granulomatosis with polyangiitis; ELISA: enzyme-linked immunosorbent assay; FACS: fluorescence activated cell sorting; FMLP: N-formyl-methionyl-leucyl-phenylalanine; GPA: granulomatosis with polyangiitis; HBSS: Hank’s balanced salt solution; HRP: horseradish peroxidase; Ig: immunoglobulin; LPS: lipopolysaccharide; MFI: mean fluorescence intensity; MPA: microscopic polyangiitis; MPO: myeloperoxidase; OD: optical density; PDGF: platelet-derived growth factor; PE: phycoerythrin; PR3: proteinase-3; S1P: sphingosine-1-phosphate; Sphk: sphingosine kinase; TMB: 3,3′-5,5′ tetramethylbenzidin; TNF: tumor necrosis factor; TNFR1: tumor necrosis factor receptor 1.

## Competing interests

The authors declare that they have no competing interests.

## Authors’ contributions

JH and YMH performed the experiments and drafted the manuscript. MC and MHZ conceived and designed the experiments. All authors read and approved the final manuscript.
